# Differences in engagement and outcomes in a postpartum hypertension monitoring program by infant NICU admission

**DOI:** 10.1002/pmf2.70310

**Published:** 2026-05-12

**Authors:** Mary Marchese, Jennifer Okunbor, Allison K. Mak, Scott Machado, Tracy L. Jackson, Nicole Konecke, William Hunt, Crystal F. Ware, Danielle Simmons, Maria Mejia‐Castillo, Methodius G. Tuuli, Alisse Hauspurg, Adam K. Lewkowitz

**Affiliations:** ^1^ Department of Obstetrics and Gynecology Women & Infants Hospital of Rhode Island/Warren Alpert Medical School of Brown University Providence Rhode Island USA

**Keywords:** hypertensive disorders of pregnancy, NICU admission, postpartum engagement, remote blood pressure monitoring

## Abstract

**Introduction:**

Remote postpartum self‐measured blood pressure (SMBP) monitoring programs improve rates of recommended BP ascertainment among individuals with hypertensive disorders of pregnancy (HDP) overall. Healthcare utilization patterns differ among individuals whose infants are admitted to the neonatal intensive care unit (NICU) and those with infants in the standard newborn nursery, but it is unclear whether this difference translates into a reduction of SMBP program engagement.

**Methods:**

Our SMBP program is offered to all postpartum individuals with HDP at our hospital. Participants were stratified by those with infants admitted to the NICU or to the newborn nursery. The primary outcome was SMBP engagement, defined as reporting ≥1 BP measurement between hospital discharge and 10 days postpartum. Secondary outcomes included the total number of SMBP measurements during the 6‐week program, hypertension (HTN)‐related Emergency Department (ED) visit or readmission, initiation of antihypertensive medication, and severe maternal morbidity (SMM), as defined by HTN‐related conditions within the CDC SMM composite. Relative risks (RRs) were calculated after adjusting for differences between study groups identified in bivariate analyses.

**Results:**

Among 2556 SMBP participants, 28% (*n* = 707) had infants admitted to the NICU. Compared to those with infants in the newborn nursery, those with infants in the NICU delivered at earlier gestational ages, were more likely to have severe preeclampsia and superimposed preeclampsia, and had higher rates of postpartum ICU admission. After adjusting for these factors, there was no significant difference in SMBP engagement (adjusted RR [aRR], 0.99; 95% confidence interval [CI], 0.89–1.10). There were no significant differences in secondary outcomes including HTN‐related ED visits, readmissions, or SMM.

**Conclusion:**

The rate of SMBP engagement was similar regardless of NICU admission and lower than has been reported in other studies. However, despite having more severe types of HDP, NICU parents had rates of postpartum HTN‐related ED visits or readmissions similar to those with infants in the newborn nursery. Collectively, these findings suggest that while remote SMBP programs may not effectively encourage adherence to recommended BP ascertainment, participation may mitigate postpartum HTN‐related morbidity in this population.

## 1 INTRODUCTION

Hypertensive disorders of pregnancy (HDP) are significant contributors to maternal morbidity and mortality [1] and postpartum readmissions [2]. To reduce these risks, the American College of Obstetricians and Gynecologist (ACOG) recommends postpartum blood pressure (BP) ascertainment for those with HDP within 7–10 days of delivery and evaluation within 72 h of delivery for those with severe HDP [3]. In individuals with HDP, remote BP monitoring has been shown to increase adherence to this recommended screening and may reduce adverse HDP‐related health outcomes, including unplanned healthcare utilization or morbidity and mortality [4]. For these reasons, close follow‐up for individuals with HDP is warranted, and remote self‐measured BP (SMBP) monitoring provides an accessible and effective option to do so by minimizing in‐person visits.

Healthcare utilization patterns differ between individuals with infants admitted to the neonatal intensive care unit (NICU) and the standard newborn nursery, perhaps because those with NICU infants may be less engaged in their own postpartum health needs due to concerns about their infant's health [5]. For example, compared to those whose infants were not in the NICU, those with NICU infants have been shown to have higher rates of postpartum emergency room visits and readmissions [6] and lower rates of receiving routine postpartum visits [7]. Individuals with HDP are at increased risk of having an infant requiring NICU admission, either via iatrogenic prematurity due to severe HDP or due to intrapartum complications [6]. However, whether having an infant admitted to the NICU is associated with different rates of engagement with remote BP programs is unclear. To fill this gap, we compared outcomes in our remote SMBP monitoring program for people with HDP among those whose infants were admitted to the NICU versus the standard newborn nursery.

## 2 MATERIALS AND METHODS

Postpartum patients with HDP are offered enrollment in our remote SMBP program. Patients are visited during their postpartum hospitalization by a nurse practitioner and community health worker and provided a free automatic BP cuff and education on SMBP, frequency of monitoring, and warning signs to seek emergency care. They are taught how to input their BP readings into the electronic medical record (EMR) to be viewed by providers and are prompted via secure electronic message or phone call from a community health worker or nurse practitioner to take their BP once daily for 6 weeks. Nurse practitioners view SMBP measurements for each participant daily during the first 6 weeks postpartum and remotely titrate antihypertensive medication as needed by calling the participant, reviewing medication changes, and providing an updated prescription. Medications initiated or up‐titrated included labetalol, nifedipine, and enalapril, per provider preference, and these medications were changed to keep more than 50% of the SMBP measurements below 140/90 mmHg.

We performed a cohort analysis of all individuals who participated in our SMBP from 2022 to 2024. Individuals were stratified by whether their infants were admitted to the NICU or the standard newborn nursery. The primary outcome was SMBP engagement, defined by reporting ≥1 BP measurement within the first 10 days postpartum per ACOG guidelines [2]. This interval was chosen because the majority of people with severe HDP remained hospitalized within 72 h of birth, so they were already receiving screening in alignment with ACOG guidelines. Secondary outcomes included total number of BP measurements reported through the 6‐week program, hypertension (HTN)‐related Emergency Department (ED) visits or hospital readmissions, initiation or dose increase of antihypertensive medications, and severe maternal morbidity (SMM), which was modified from the Centers for Disease Control and Prevention composite [8]. Baseline characteristics were compared using Wilcoxon rank‐sum tests for continuous variables and Fisher's exact tests for categorical variables. Adjusted relative risks (aRRs) were estimated using generalized linear models, controlling for differences between groups identified in bivariate analyses.

## 3 RESULTS

Among 2556 patients enrolled in the SMBP, 707 (27.7%) had infants admitted to the NICU (Table 1). Those with infants in the NICU delivered at an earlier gestational age than those with infants admitted to the nursery (*p* < 0.0001). Types of HDP diagnoses differed between groups, with severe forms of HDP more common in those with infants admitted to the NICU compared to those with infants admitted to the nursery (Table 1). Other notable differences between groups include mode of delivery, rate of maternal ICU admission, and primary maternal language (Table 1).

**TABLE 1:** Demographic and delivery characteristics by infant NICU admission status.

	All enrolled *N* = 255	NICU *N* = 707	Nursery *N* = 1849	*p* value
Age in years	32 [27–35]	32 [28–36]	31 [27–35]	0.05
Primary insurance type				0.34
Private	1404 (54.9%)	373 (52.8%)	1031 (55.8%)	
Medicaid/Medicare	1139 (44.6%)	331 (46.8%)	808 (43.7)	
None	10 (0.4%)	2 (0.3%)	8 (0.4)	
Race				0.51
White only	1446 (56.6%)	415 (58.7%)	1031 (55.8%)	
Black only	463 (18.1%)	125 (17.7%)	338 (18.3%)	
Other race/mixed race	610 (23.9%)	156 (22.1%)	454 (24.6%)	
Unknown	37 (1.5%)	11 (1.6%)	26 (1.4%)	
Language				0.03
English	2092 (82.0%)	597 (84.8%)	1495 (81.0%)	
Spanish	384 (15.1%)	85 (12.1%)	299 (16.2%)	
Other	74 (2.9%)	22 (3.1%)	52 (2.8%)	
HDP diagnosis at discharge	2064 (86.3%)	614 (89.8%)	1450 (84.9%)	.002
Gestational hypertension	931 (36.5%)	165 (23.4%)	766 (41.5%)	<.0001
Preeclampsia without severe features	327 (12.8%)	70 (9.9%)	257 (13.9%)	.007
Preeclampsia with severe features	798 (31.3%)	349 (49.4%)	449 (24.3%)	<.0001
Eclampsia	10 (0.4%)	5 (0.7%)	5 (0.3%)	0.15
Chronic hypertension with superimposed preeclampsia	216 (8.5%)	105 (14.9%)	111 (6.0%)	<.0001
Chronic hypertension alone	327 (13.7%)	70 (10.2%)	257 (15.1%)	0.002
Gestational age at delivery (weeks)	38.0 [37.00–39.29]	35.1 [33.21–37.27]	38.6 [37.43–39.43]	<0.0001
Mode of delivery				<0.0001
Vaginal	1248 (48.9%)	196 (27.8%)	1052 (57.0%)	
Cesarean section	1305 (51.1%)	510 (72.2%)	795 (43.0%)	
Maternal ICU admission within seven days of delivery	12 (0.5%)	8 (1.1%)	4 (0.2%)	0.005
Duration of delivery hospitalization (days)	4 [3–5]	5 [4–7]	4 [3–5]	<0.0001

*Note*: Data are *n* (%) or median [interquartile range] unless otherwise noted.

Abbreviations: HDP, hypertensive disorders of pregnancy; ICU, intensive care unit; NICU, neonatal intensive care unit.

There was no difference identified between those with infants admitted to the NICU versus nursery in the rate of SMBP engagement within the first 10 days postpartum (NICU: *n* = 367 [52%] vs. nursery: *n* = 865 [47%]; aRR, 0.97; 95% confidence interval [CI], [0.86, 1.08]).

Similarly, the number of BP measurements reported per patient did not significantly differ between those with infants in the NICU or nursery (NICU: *n* = 10.3 vs. nursery: *n* = 9.0; *β* = −1.17 [−2.74, 0.40]). Though the proportion of people who had an HDP‐related ED presentation or readmission was lower among those with infants admitted to the NICU versus those in the nursery (*n* = 10.8% vs. *n* = 16.6%), after adjusting for confounders, this difference was not significant (aRR, 0.78; 95% CI, [0.60–1.03]). There were also no differences between groups in remote medication initiation or titration, or overall rates of SMM (Table 2).

**TABLE 2:** Outcomes by infant NICU admission among all enrolled.

All enrolled						
	All enrolled *N* = 2556	NICU *N* = 707	Nursery^b^ *N* = 1849	*p* value	Relative risk/*β* (95% CI)	Adjusted RR/Adjusted *β* (95% CI)^a^
Primary outcome						
Reporting a BP within first 10 days postpartum	1232 (48.2%)	367 (51.9%)	865 (46.7%)	0.02	1.11 (1.02, 1.20)	0.97 (0.86, 1.08)
Secondary outcomes						
Total number of BPs reported	9.4 (14.4%)	10.3 (14.4%)	9.0 (14.4%)	0.05	1.28 (0.00, 2.49)	−1.35 (−2.89, 0.18
BP med initiated	758 (29.8%)	267 (37.9%)	491 (26.7%)	<.0001	1.42 (1.25, 1.60)	1.16 (0.99, 1.37)
Any HTN‐related postpartum readmission or ED visit	383 (15.0%)	76 (10.8%)	307 (16.6%)	<.0002	0.64 (0.51, 0.81)	0.77 (0.55, 1.06)
HTN‐related ED visit	372 (14.7%)	73 (10.3%)	303 (16.4%)	<.0001	0.63 (0.49, 0.80)	0.72 (0.52, 1.02)
HTN‐related readmission	238 (9.3%)	39 (5.5%)	199 (10.8%)	<.0001	0.51 (0.37, 0.71)	0.72 (0.45, 1.16
Severe maternal morbidity^c^	316 (12.6%)	129 (18.3%)	189 (10.2%)	<.0001	1.79 (1.45, 2.19)	1.33 (1.00, 1.75)
Stroke	2 (0.1%)	1 (0.1%)	1 (.1%)	0.48		
Seizure/eclampsia	13 (61.5%)	5 (0.7%)	8 (0.4%)	0.37		
Acute fatty liver of pregnancy	2 (0.1%)	1 (0.1%)	1 (0.1%)	0.48		
Posterior reversible encephalopathy syndrome	1 (0.04%)	0 (0%)	1 (0.1%)	1.0		
Pulmonary edema	46 (1.8%)	21 (3.0%)	25 (1.4%)	0.01		
Heart failure	1 (0.04%)	1 (0.1%)	0 (0.0%)	0.28		
HELLP	48 (1.9%)	28 (4.0%)	20 (1.1%)	<.0001		
Placental abruption	20 (0.8%)	17 (2.4%)	3 (0.2%)	<.0001		
Postpartum hemorrhage	192 (7.5%)	61 (8.6%)	131 (7.1%)	0.21		
Acute kidney injury	3 (0.1%)	3 (0.1%)	0 (0.0%)	0.02		
Transaminitis	19 (0.7%)	7 (1.0%)	12 (0.7%)	0.44		

*Note*: Data presented as *n*(%) or mean (SD) unless noted.

Abbreviations: BP, blood pressure; CI, confidence interval; ED, Emergency Department; HELLP, hemolysis, elevated liver enzymes, low platelets; HTN, hypertension; NICU, neonatal intensive care unit; RR, relative risk; SMM, severe maternal morbidity.

^a^ Adjusted for age, language, mode of delivery, gestational age at delivery, length of delivery hospitalization, and type of hypertensive disorder.

^b^ Reference group.

^c^ RR not calculated for individual metrics within the SMM composite.

## 4 DISCUSSION

This study provides the first data on SMBP program engagement among postpartum people with HDP who have infants admitted to the NICU. Though the rate of engagement is lower in our cohort than in other programs [4, 9], the rate of engagement did not differ between groups, suggesting our SMBP program engages NICU and non‐NICU parents similarly.

Given that patients with NICU involvement have been noted to have less engagement in postpartum care [7], our findings suggest that SMBP programs for those with HDP may engage patients with and without NICU involvement equally. Furthermore, despite having a higher prevalence of severe HDP and ICU admission than those who have infants in the nursery, the rate of unplanned healthcare utilization for HTN‐related indications among those with infants in the NICU was similar. It is possible that this mitigation is not through achievement of BP ascertainment goals but possibly through enhanced education about distinguishing between common symptoms of HDP and warning signs for which emergency care is warranted. This finding supports prior literature suggesting that SMBP program participation may mitigate postpartum HDP‐related complications [4, 9].

This study has many strengths. Participants in our SMBP program are diverse ethnically and racially and include both English and Spanish speakers, which adds generalizability to the findings. In addition, all data were collected prospectively, adding robustness to our results. Nevertheless, this study is not without limitations. First, the study population is derived from a single academic hospital, which may limit generalizability to community hospitals. Second, our hospital is the major regional referral center, and maternal transfers regularly occur to reunite mothers with their infants who have been transferred to our NICU. Our SMBP program is available to all postpartum people, regardless of their delivery hospital. Thus, it is possible that people who delivered at another hospital may have preferred postpartum in‐person BP ascertainment with their primary obstetricians instead of SMBP engagement. Lastly, our study viewed NICU admission as a homogeneous exposure and had lower rates of engagement compared to what is reported in the literature [9]. Further research in a larger population is warranted to assess whether HTN‐related outcomes among SMBP participants differ by length of infant hospitalization, which was not included in this study design, particularly among those whose infants remain in NICU for weeks or more. Future investigation into additional factors associated with SMBP engagement can promote improved understanding of postpartum health care utilization and outcomes among patients experiencing NICU admission for their infants. This study did not evaluate differences in achievement of goals for BP between the NICU and newborn nursery populations, and thus presents an opportunity for future research to evaluate differences in efficacy between these two populations. One potential strategy to optimize engagement for the NICU population includes strengthening collaboration with our NICU‐specific social work team that performs outreach with each parent of an NICU‐admitted infant to incorporate our SMBP as part of wraparound services for those with HDP. Additionally, we anticipate that adherence in this population will be increased in the more technology‐enabled SMBP program that we are currently studying [10]. This new program provides notifications reminding participants to take their BP, automatically transfers the BP into the EMR, and provides feedback based on the BP value to the providers.

In conclusion, in our remote SMBP program, there was no difference in engagement or unplanned healthcare utilization for HTN‐related etiologies among people whose infants were versus were not admitted to the NICU.

## CONFLICT OF INTEREST STATEMENT

A.K.L. and A.H. served as paid consultants for Sonio AI in 2024. All other authors declare no conflicts of interest.

## ETHICS STATEMENT

Informed consent for participation was obtained. This study was approved by the Care New England Institutional Review Board (2187816, Approved May 20, 2024).

## Data Availability

The data that support the findings of this study are available from the corresponding author upon reasonable request.
